# Supplementary material for: Mutations in PCYT2 disrupt etherlipid biosynthesis and cause a complex hereditary spastic paraplegia
Source: Brain. 2019 Oct 22;142(11):3382–97. doi: 10.1093/brain/awz291 (PMC6821184; doi:10.1093/brain/awz291)
Supplement: awz291_Supplementary_Data [file awz291_supplementary_data.zip › awz291-Suppl_data/Supplementary_Data4.zip!/Supplementary information 4 - plasma lipidomics.zip!/Plasma lipidomics/Control vs Patient1/Statistical plots and data/Heatmap VIP-score bigger than 1.pdf]

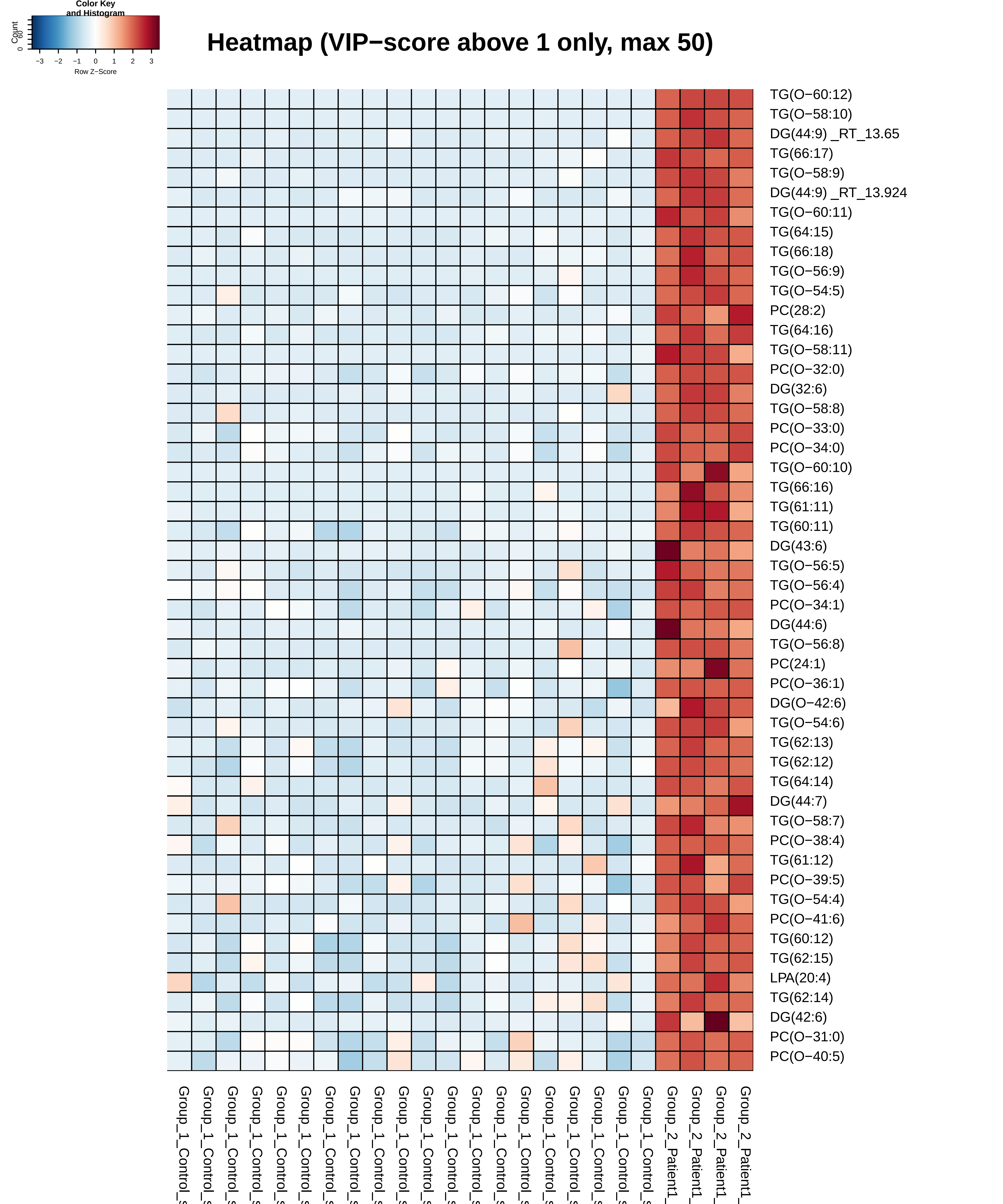
Color Key
and Histogram
Count
0
60
−3
−2
−1
0
1
2
3
Row Z−Score
Heatmap (VIP−score above 1 only, max 50)
TG(O−60:12)
TG(O−58:10)
DG(44:9) _RT_13.65
TG(66:17)
TG(O−58:9)
DG(44:9) _RT_13.924
TG(O−60:11)
TG(64:15)
TG(66:18)
TG(O−56:9)
TG(O−54:5)
PC(28:2)
TG(64:16)
TG(O−58:11)
PC(O−32:0)
DG(32:6)
TG(O−58:8)
PC(O−33:0)
PC(O−34:0)
TG(O−60:10)
TG(66:16)
TG(61:11)
TG(60:11)
DG(43:6)
TG(O−56:5)
TG(O−56:4)
PC(O−34:1)
DG(44:6)
TG(O−56:8)
PC(24:1)
PC(O−36:1)
DG(O−42:6)
TG(O−54:6)
TG(62:13)
TG(62:12)
TG(64:14)
DG(44:7)
TG(O−58:7)
PC(O−38:4)
TG(61:12)
PC(O−39:5)
TG(O−54:4)
PC(O−41:6)
TG(60:12)
TG(62:15)
LPA(20:4)
TG(62:14)
DG(42:6)
PC(O−31:0)
PC(O−40:5)
Group_1_Control_
Group_1_Control_
Group_1_Control_
Group_1_Control_
Group_1_Control_
Group_1_Control_
Group_1_Control_
Group_1_Control_
Group_1_Control_
Group_1_Control_
Group_1_Control_
Group_1_Control_
Group_1_Control_
Group_1_Control_
Group_1_Control_
Group_1_Control_
Group_1_Control_
Group_1_Control_
Group_1_Control_
Group_1_Control_
Group_2_Patient1_
Group_2_Patient1_
Group_2_Patient1_
Group_2_Patient1_
